# Case of Dropsy, in Which Large Doses of Kino Appeared to Be of Essential Service

**Published:** 1827-02

**Authors:** 

**Affiliations:** Elgin, in a Letter to Sir James M'Grigor.


### DROPSY.

*Case of Dropsy, in which large Doses of Kino appeared to be of essential Service.* Communicated by Dr. Paul, of Elgin, in a Letter to Sir James M'Grigor.

When I did myself the honour of writing to you in February relative to your friend Colonel G——, of the Royal African Corps, I mentioned that, as the remedies usually employed in the disease under which he laboured had entirely failed, we had adopted a plan of treatment founded on a principle very different from that on which the cure is generally conducted; the result of which I promised to lay before you. I would have done so long ere this time, if you had not had an opportunity of seeing the Colonel in London, and of knowing every thing from himself, as I thought, which was worthy of being communicated. Colonel G—, however, assures me that he could only give you a very imperfect idea of his case; and, as you felt an interest in knowing what had been of use to him, and expressed some astonishment on hearing his own account of it, he is particularly anxious that I should give you a more circumstantial one.

The Colonel, as you know, returned to Elgin in October 1825, from the coast of Africa, where his health had suffered much from repeated attacks of fever. At that time emaciation had made alarming progress. He had constant irritative fever; pulse varying from 110 to 120; features very sharp, I may almost say hippocratic; great prostration of strength; abdomen tumid, evidently containing fluid; considerable tenderness on pressure over the hepatic region; anasarca of the feet and ankles; perspirations in the morning; great irregularity of the bowels; for the most part they were too open, but the discharges were not always fluid; he made about the usual quantity of urine, it was of a dark brown colour.

When I first saw him, he was slightly under the influence of mercury. The mercurial action was kept up in the same gentle manner, by rubbing in small quantities of the ointment, with the view of restoring healthy action in the liver, and also of promoting the absorption of the effused fluid. Strict attention was paid to the bowels, both to carry off vitiated secretions and to quiet irritability. Powerful frictions over the abdomen and along the spine, with the hand and flesh-brush, using a stimulating liniment. An attempt was made at the same time to act, if possible, powerfully on the kidneys by diuretics, and the most powerful of this class of medicines were exhibited,—viz. Tinct. Digitalis, Squills, Spiritus Ætheris Nitrici, Acet. Potassæ, &c. variously combined. Broom-tops were thought of, but there appeared to be too great relaxation of the system, and of the intestines in particular, for their exhibition.

We persevered in the manner now specified till the 26th of December, when he had become worse in every respect: the fluid in the belly had accumulated so much, that it was quite impossible to think of affording relief but by tapping. Your friend Dr. Skene, who saw him at this time, concurred in the propriety of having the fluid drawn off, giving at the same time a very unfavourable prognosis. On the 26th December he was tapped, and eighteen pints of fluid drawn off. The diuretics were again administered with the most scrupulous attention. The mercury was discontinued on account of increasing debility. The pain in the side on pressure was still complained of. The diuretics disturbed the stomach exceedingly, and had no effect on the urinary secretion. The water accumulated again very rapidly in the belly, and his ankles were much swelled. He appeared now to be in the most imminent peril; indeed, so great was the prostration of strength, that we were afraid that tapping might bring on fatal syncope. It was, however, done on the 24th January; taking the precaution of stopping the discharge seven or eight times, and giving wine: sixteen pints were discharged. During the time it was flowing, he complained a good deal of uneasiness about the right side of the thorax. For several hours after the operation, he appeared to be so exhausted that he could not be removed from the sofa. For two or three days we were afraid that he was sinking. His strength again began to rally; the fluid in the belly accumulated as rapidly as ever.

The diuretics having been fully tried, and without producing the least effect, it was thought useless to continue them longer, particularly as they disturbed the stomach. It occurred to me that some advantage might be derived from acting on the following principle: to prevent, if possible, the further accumulation of the fluid, and to trust solely to the natural actions of the body for the absorption of what was effused. On the 3d of February, the Tincture of Kino was prescribed, and port wine substituted for sherry. For the first few days, he took four drachms of the tincture daily in divided doses, in port wine; and then he took daily one ounce, and fully half a bottle of port, till the 1st of April; and since that time he has taken one ounce only in the three days.

When he began to take the kino, he was nearly as full as before tapping: after taking it for eight or ten days, symptoms of amendment began to manifest themselves. His appetite improved; he became stronger; pulse was less frequent. The abdominal swelling was found, by exact measurement, to be stationary; and very soon it evidently decreased, so that, at the time the dose of the tincture of kino was reduced, he was able to go some distance from home.

When I first saw him after his arrival from London, I was forcibly struck with the improvement which had taken place since his departure; and, although there may be still some fluid in the abdomen, it is not, I think, unreasonable to expect that it will be altogether removed, when we reflect on what has taken place.—

He has taken, since he has been under my care, every eight or ten days, at night, three grains of Calomel, and next morning half an ounce of Tinctura Rhei, to carry off vitiated and to promote healthy secretions.

Forgetful, as we often are, of the powers of nature in effecting salutary changes of disease, and wishful to impute them to the agency of remedies, yet in this case it is fair to admit, I think, that the kino was of essential service. In corroboration of this opinion, I may mention that, whilst he was in London, he discontinued the kino for a time, and his belly became so tumid that he could not button his coat; but, after using the kino for some time, it again decreased. The theory of its operation in dropsical effusions, as far as I can judge, is that, when taken into the circulating system, it acts as a tonic, promoting the contractility of the blood-vessels, without stimulating them materially to increased action, thereby preventing superabundant exhalation; by which means the absorbents remove more than what is effused. In Colonel G—'s case, by its tonic operation on the intestinal canal, the alvine discharges were diminished in frequency, but nothing like constipation was produced.

From the effect which the tincture of kino has had in controlling the dropsical effusions in this case, I am induced to think that it will be found, on further trial, to be a valuable medicine in some of the varieties of dropsy. When it depends on any inflammatory condition of serous membranes, or when there is much visceral disease, it is not likely that kino would be of use; yet, in Colonel G—'s case, there was considerable affection of the liver. I am not aware that kino has ever been given in such large doses, and with the same views, as in the present case.

*Elgin; November 17th*, 1826.

